# Redefining language networks: connectivity beyond localised regions

**DOI:** 10.1007/s00429-024-02859-4

**Published:** 2024-11-18

**Authors:** Stephanie J. Forkel, Peter Hagoort

**Affiliations:** 1 Donders Institute for Brain Cognition Behaviour, Radboud University, Nijmegen, The Netherlands; 2 Max Planck Institute for Psycholinguistics, Nijmegen, The Netherlands; 3 Brain Connectivity and Behaviour Laboratory, Sorbonne Universities, Paris, France; 4 Centre for Neuroimaging Sciences, Department of Neuroimaging, Institute of Psychiatry, Psychology and Neuroscience, King’s College London, London, UK

**Keywords:** Language, Networks, Systems, White matter, Connectome, Interindividual variability

The study of the neurobiology of language is entering an exciting era, marked by new perspectives and methodologies that go beyond traditional localisationist approaches. As we deepen our understanding of how language functions in the brain, it is clear that no single cortical area can fully account for the complexity of language processing. Rather, language processing requires dynamic interactions across widespread neural networks involving cortical regions and subcortical structures embedded into a network of white matter pathways (Thiebaut de Schotten and Forkel 2022). Moreover, the execution of our language skills (e.g. speaking, listening, reading) relies on robust connections to various cognitive systems, including memory and executive control (Hagoort 2017). Processing of language is not only based on the retrieval of lexical knowledge but also interacts with multiple other sources of information (e.g. co-speech gestures, emotional prosody, the conversational setting) that co-determine the interpretation of the linguistic input and co-modulate the characteristics of the spoken output. It is also important to realise that communication through language is more than exchanging propositional content. With a linguistic utterance, the speaker aims to change the state of mind of a listener or prompt them to take action. For example, the statement “It is cold in here” is often an implicit request to do something about it (e.g. to close the window). The inference from coded meaning to speaker meaning depends on the contribution of the so-called Theory of Mind network in the brain (Hagoort and Levinson 2014). In short, commanding a language goes beyond core areas for retrieving and combining linguistic information. It requires additional contributions from multiple cortical and subcortical networks.

In this *Brain Structure & Function collection* titled *Language Systems*, we invited contributors to explore language from diverse perspectives, across brain states, and encompassing languages beyond English. This collection explores how the brain orchestrates linguistic processing through multiple interconnected systems. The research presented here highlights that no singular brain region or pathway is responsible for language. Consequently, the notion of a single, unified language area or network is a misconception (Hagoort 2019; Forkel et al., 2021; Thiebaut de Schotten and Forkel 2022). These studies highlight the complexities of language processing by examining the structural and functional architectures that support it, offering fresh insights into how diverse brain structures collaborate to facilitate language.

By employing advanced methodologies such as neuroimaging, computational modelling, and large-scale data analyses, the papers in this collection provide a comprehensive view of the multifaceted nature of language. We aim to shift the dialogue from a narrow, localised language perspective to one that fully embraces its dynamic and distributed character, recognising the importance of cortical, subcortical, and connectional contributions to linguistic function (Fig. 1).

**Fig. 1:**
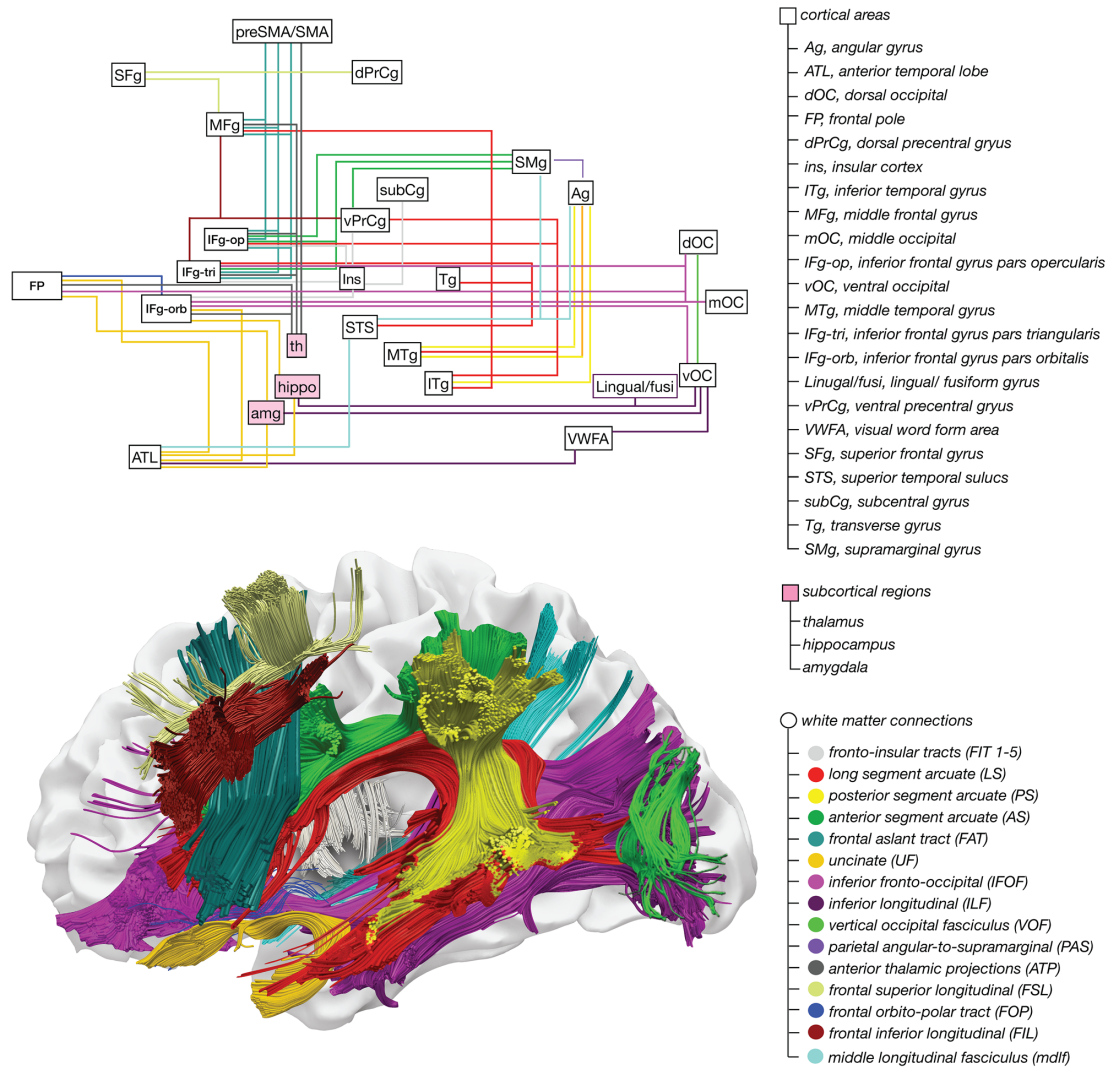
Structural connectivity of language-relevant associative white matter pathways and their cortical and subcortical termination and relay areas. Many of these connections have been clinically validated through neurosurgical techniques, lesion studies, and direct electrical stimulation, underscoring their relevance in language processing. Additional pathways are included for completeness; while their connections to critical regions (e.g., fronto-insular tracts) suggest potential significance, they await further functional validation

## Key contributions

A central piece revisits Wernicke’s 150-year-old model, offering a contemporary review of its evolution and relevance. Roelofs integrates data from patient studies and computational models, explaining how Wernicke’s reflex arc concept has been updated with mechanisms such as attentional control (Roelofs 2024). This perspective highlights how our understanding of language processing has advanced and continues to evolve with novel insights and new data.

Current functional language localisation protocols are often univariate, treating each small unit of brain volume as independent. Functional magnetic resonance imaging (*f*MRI) is a notable example, where researchers commonly compare neural responses to sentences with pseudowords [pronounceable nonwords; e.g. (Fedorenko et al. 2024, 2010)]. While this method consistently activates patterns in peri-sylvian regions involved in the syntactical processing of sentences, it is a less effective paradigm to identify activity in extra-sylvian areas related to semantics and word meanings. Graves et al. propose that using a multivariate region of interest (ROI) approach—targeting brain areas consistently active across participants—more effectively captures brain functions tied to meaning (Graves et al. 2023). Their work highlights vital structures involved in semantics, such as the frontal gyri (Binder et al. 2009), bilateral anterior superior temporal gyrus (Lambon Ralph et al. 2010), left middle temporal gyrus (Turken and Dronkers 2011), angular gyrus (Seghier 2013), and the posterior cingulate cortex (Leech and Sharp 2014). Combining univariate and multivariate approaches could provide a more complete understanding of how the brain processes different linguistic features of language.

How different types of language usage—for example, emotive and referential or comprehension and production—are supported by specialised neural networks reflects the multifaceted nature of language processing. It demonstrates the brain’s dynamic ability to adapt to diverse linguistic demands. Ma et al. explore this by examining how the brain processes emotive and referential language, revealing that the right temporoparietal junction is activated for emotive language, while referential language primarily engages left hemisphere networks (Ma et al. 2022). This finding expands our understanding of how distinct neural pathways handle emotional versus factual language. Similarly, Roos et al. introduce the Concise Language Paradigm (CLaP), which investigates comprehension and production simultaneously using electrophysiological methods (Roos et al. 2024). Their study highlights the brain’s delicate balancing act between understanding and generating language, focusing on alpha–beta oscillatory dynamics. This novel approach provides fresh insights into the neural mechanisms that support concurrent language functions, offering a more nuanced view of the brain’s flexibility in processing language. Together, these studies underscore the brain’s capacity to adapt specialised networks for different linguistic functions, enriching our understanding of how language systems operate.

Another critical focus is interindividual variability in brain anatomy (i.e. neurovariability) and its impact on language function (Forkel et al. 2021). One highly variable structure is the transverse temporal gyrus (TTG), commonly known by its eponym Heschl’s gyrus, which exhibits considerable interindividual variability (Henderson et al. 2023; Marie et al. 2015). Observations of such anatomical variability are generally well-documented e.g. (Amunts et al. 1999; Caspers et al. 2006; Fornito et al. 2006; Ono et al. 1990), and the variability of the transverse gyrus might point toward the intricate nature of auditory processing and speech features. Ramoser et al. examine the multiplication pattern of the bilateral TTG in relation to language ability (Ramoser et al., 2024). Their observations suggest that fewer gyri in the right hemisphere and a larger surface area in the first right transverse gyrus and the second left are associated with language aptitude. Eckert et al. further demonstrate that a duplicated transverse gyrus correlates with lower phonological decoding abilities, highlighting how structural differences in the transverse gyrus can influence language aptitude and broader cognitive abilities like reading and auditory processing (Eckert 2024).

Looking at the white matter involved in a spelling-to-dictation task, Sagi et al. report that high-performing in spelling significantly correlated with the left inferior longitudinal fasciculus (Fig. 1; Sagi et al. 2024). In contrast, low-performing in spelling correlated with the right superior longitudinal fasciculus. These results suggest that high performers rely more on lexical-orthographic processes, while low performers depend on phoneme-to-grapheme conversion.

The role of subcortical structures in language processing is also gaining recognition, with clinical studies providing critical insights. Patients with thalamic strokes, for example, often exhibit language impairments with a predominance of lexical-semantic difficulties, underscoring the thalamus’ importance in modulating communication between cortical areas involved in language (Fritsch et al. 2022; Rangus et al. 2024). Similarly, an estimated 90% of people with Parkinson’s Disease experience difficulties with their voice and speech, known as hypokinetic dysarthria, meaning that they speak softly, with a monotonous pitch, slurred articulation, and abnormal speech rates (Ramig et al. 2008). After undergoing deep brain stimulation (DBS) of the subcortical grey matter in the basal ganglia (thalamus, caudate, putamen, globus pallidus), DBS is highly effective in alleviating the cardinal motor symptoms of PD; however, it has mixed outcomes when it comes to speech. Some patients experience improvements in their speech with DBS (Pinto et al. 2005), while others experience their speech worsening over time (Bronstein et al. 2011; Tripoliti et al. 2011, 2014; Wertheimer et al. 2014). The reason for these conflicting outcomes, however, remains unclear. Bulut and Hagoort’s meta-analysis of the healthy brain emphasises the bilateral thalamus’ role in cortical-thalamo-cerebellar loops, revealing its coactivation with frontal and temporal regions and subcortical areas like the basal ganglia and cerebellum during language tasks (Bulut and Hagoort 2024). Bulut & Hagoort suggest that cortico-subcortical-cerebellar-cortical loops modulate and fine-tune information transfer within the bilateral frontotemporal cortices during language processing, especially during production and semantic operations, but also other language (e.g. syntax, phonology) and cognitive operations (e.g. attention, cognitive control). The cerebellum has recently been mapped in detail for its contributions to language (Turker et al. 2023). Petríková et al., therefore, targeted the cerebellum by transcranial direct current stimulation (tDCS) to assess and compare the contribution of the cerebellar processing to automatic and controlled retrieval of words in healthy adults (Petríková et al. 2023). They report that cerebellar tDCS facilitated the retrieval of sequentially related words in free-associative word chains. Still, it did not affect tasks requiring semantic control, such as inhibiting unrelated words or switching flexibly between retrieval rules. This work broadens our understanding of how language networks extend beyond the cortico-centric view, highlighting the significant contributions of subcortical and cerebellar structures to complex language functions.

Additionally, this collection goes beyond studies of English-speaking populations to explore how diverse languages engage brain networks differently. Seghier and Boudelaa’s review of the neuroimaging literature on Arabic reading, for example, demonstrates how the unique orthographic features of Arabic activate distinct neural pathways, challenging established neuroanatomical models of reading (Seghier and Boudelaa 2024). Kumar et al. examine the brain’s processing of Sanskrit verse using functional imaging and show how it adapts to ancient languages’ complex linguistic and rhythmic structures (Kumar et al., 2024). Quiñones et al. explore the neuroplasticity of the bilingual brain, revealing how the coexistence of multiple languages reshapes the structure and function of neural networks, mainly through the recruitment of broader bilateral basal ganglia-thalamo-cortical circuits (Quiñones et al. 2024). Together, these studies emphasise the importance of considering diverse languages and their neural mechanisms to fully understand the brain’s language networks.

The clinical studies in this collection focus on language disorders and the neuroanatomy underlying language recovery, especially following stroke or surgical interventions. Stockbridge et al. investigate white matter integrity in subacute post-stroke aphasia patients (Stockbridge et al. 2024). An atlas-based diffusion tensor imaging tractography analysis revealed that recovery is closely linked to changes in critical white matter tracts, including the arcuate and superior longitudinal fasciculi. Their findings suggest that microstructural integrity can serve as a predictor of language recovery. Similarly, Kram et al. explore surgical risk stratification in glioma patients by examining pre- and postoperative changes in language-related tracts, such as the arcuate fasciculus and inferior fronto-occipital fasciculus (Fig. 1) (Kram et al. 2024). Their work underscores the importance of tract integrity in predicting post-surgical language outcomes and provides valuable insights for clinical decision-making.

In summary, this collection offers a multifaceted exploration of language systems, highlighting how diverse methods, languages, and populations provide a more complete picture of how language networks operate across the entire brain. This collection extends beyond classical models and contributes valuable insights into healthy and disordered language processing.

## A call for a shift in thinking

The traditional approach of pinpointing “where” language happens in the brain is increasingly seen as an oversimplification. Language is not an isolated function confined to a few discrete regions; rather, it emerges from a web of dynamic, interconnected systems. These systems adapt, collaborate, and respond based on the task, whether speech production, comprehension, or reading. The contributions in this issue collectively challenge a cortico-centric localisationist view, arguing for a more integrated, systems-level approach.

Language processing is now better understood as relying on the interactions between various neural networks, including cortical and subcortical regions. For example, recent studies have highlighted how subcortical structures like the thalamus are integral to language processing—working in concert with cortical areas and connected by the brain’s white matter (Fig. 1). This perspective represents a significant shift from the old models focused on individual language “centres,” such as Broca’s and Wernicke’s areas. Instead, the results reported in this collection show that language processing is based on the complex interactions of multiple systems spanning large parts of the brain.

This evolving understanding opens new approaches in fundamental research and clinical applications. Moving beyond a localisation framework (Catani et al. 2012; Noble et al. 2024), we can explore language in its full complexity—considering how language interacts with memory, cognitive control, and emotions. This shift in thinking promises to improve our theoretical models of language and how we approach language disorders, potentially moving toward treatments that address language’s dynamic and distributed nature in the brain. As these contributions illustrate, embracing this broader, network-based perspective will provide a richer and more accurate understanding of how language operates across diverse populations and in different linguistic contexts.

## Moving forward

This network perspective underscores the flexibility of the brain’s language systems, adapting to different environments, languages, tasks, and individual anatomical differences.

As our field continues to evolve, researchers and clinicians alike must embrace the complexity inherent in language processing. Traditional, reductionist language models—focused on singular brain regions—are no longer sufficient to explain the rich diversity of linguistic function nor the clinical reality in neurological and neurosurgical patients. The studies in this issue demonstrate the importance of methodologies that capture the broader picture, such as multivariate neuroimaging, computational modelling, and large-scale data analysis embedded into theoretical solid conceptualisations. Such techniques allow us to see how language is processed across distributed networks, providing new insights from language development in the early years to recovery after brain injury in senior years.

By understanding that language is shaped by the interactions of multiple brain networks, we can advance theoretical models and clinical applications. This shift in understanding also brings new potential for enhancing therapies for language disorders, offering a more tailored approach that considers the brain’s broader role in language function and recovery.

Moreover, this network-based perspective deepens our scientific understanding and fosters interdisciplinary collaboration. It encourages researchers to draw from cognitive neuroscience, linguistics, psychology, and computer science. These insights will likely lead to new, multimodal, multidimensional and dynamic language models and more effective treatments for individuals with language impairments.

## Data Availability

The data used for the figure in this editorial is based on tractography data from the Human Connectome Project (HCP). Access to the original HCP dataset is available through the HCP database (https://www.humanconnectome.org) in compliance with HCP data use agreements. The processed data is available on the lab’s websites at https://www.bcblab.com and https://www.stephanieforkel.com/opendata.
